# A prospective randomized study to compare standard versus intensive training strategies on long-term improvement in critical care ultrasonography proficiency

**DOI:** 10.1186/s12909-022-03780-2

**Published:** 2022-10-24

**Authors:** Reina Suzuki, Mio Kanai, Kazumasa Oya, Yohei Harada, Ryohei Horie, Hiroshi Sekiguchi

**Affiliations:** 1 Division of Pulmonary and Critical Care Medicine, Mayo Clinic, Rochester, MN 55905 USA; 2 Department of Critical Care Medicine, Mayo Clinic, Phoenix, AZ 85054 USA

**Keywords:** Critical care, Fellowship and scholarship, Medical education, Point-of-care testing, Ultrasonography

## Abstract

**Background:**

Critical care ultrasonography (CCUS) has become a daily diagnostic tool for intensivists. While the effective training measures for ultrasound novices are discussed widely, the best curriculum for the novices to retain a long-term proficiency is yet to be determined.

**Methods:**

Critical care medicine fellows who underwent an introductory CCUS workshop were randomly allocated into the standard training (ST) or the intensive training (IT) group. The IT group received an 8-h training besides the standardized fellowship education that the ST group received. Participant improvement in CCUS proficiency tests (maximum score, 200) after a 6-month training intervention was compared between the groups. CCUS examinations performed in patient care were observed over 2 years.

**Results:**

Twenty-one fellows were allocated into the ST (*n* = 10) or the IT (*n* = 11) group. No statistically significant difference was observed in the median (interquartile range [IQR]) improvement in CCUS proficiency tests between the ST group and the IT group: 18 (3.8–38) versus 31 (21–46) (*P* = .09). Median (IQR) test scores were significantly higher in postintervention than preintervention for both groups: ST, 103 (87–116) versus 124 (111–143) (*P* = .02), and IT, 100 (87–113) versus 143 (121–149) (*P* < .01). Participating fellows performed 226 examinations over the 2 years of observation.

**Conclusions:**

Fellows improved their CCUS proficiency significantly after 6-month training intervention. However, an additional 8-h training did not provide further benefits.

**Supplementary Information:**

The online version contains supplementary material available at 10.1186/s12909-022-03780-2.

## Introduction

Critical care ultrasonography (CCUS) is goal-oriented ultrasonography performed and interpreted by intensivists at the bedside [1–3]. It has been used increasingly in the careof critically ill patients. Previous studies have demonstrated that the clinical utility of ultrasonography depends on the experience and skills of the operator [4–12]. Operator training is essential for ultrasonographic examination to be a beneficial tool, and general agreement exists that training must include basic knowledge of ultrasonography physics and supervised image acquisition and interpretation practice [2, 7, 8, 13–17]. Several studies have shown that basic CCUS skills can be taught efficiently in an organized workshop with the primary aim of short-term improvement [18–22].

Long-term sustainment or improvement in procedures is a mandatory but challenging mission for practicing physicians. For instance, intensivists undergo advanced cardiovascular life support certification every 2 years; yet, cardiovascular resuscitation knowledge and skill start to regress before recertification [23–28]. Especially, psychomotor skills represented in chest compressions are more difficult to retain than resuscitation knowledge [24, 29]. Similarly, a recent study on cardiac ultrasonography reported that examination skills deteriorate markedly within 2 years of nonuse [30]. A study on learning trajectory of novice ultrasound learners revealed that the image acquisition skill decayed as early as 4 weeks from the initial training [31]. Despite concern about sustainment of CCUS skills, a limited number of reports describe a medium to long-term outcome of CCUS educational effort. A study evaluating a 14-week simulation-based training for fellows resulted in comparable or greater knowledge and confidence in CCUS, relative to experts and apprentice learners [32]. Another study demonstrated that a 1-year training curriculum consisting of didactic lectures and subsequent self-paced scanning resulted in improved time to attain adequate cardiac ultrasonography images [33].

Our crucial goal was to develop a high-quality, continuous ultrasonography education program for intensivists. In the present study, we aimed to investigate the effect of a CCUS refresher training program for critical care medicine fellows who underwent an introductory workshop at the beginning of their academic year. Fellows were randomly assigned to intensive training (IT) or standard training (ST). We hypothesized that the IT group, who received CCUS refresher training, would improve in CCUS proficiency significantly more than the ST group, who received the standard CCUS training outlined in the fellowship education curriculum at our institution. Our primary end point was improvement in CCUS proficiency test scores at completion of a 6-month study intervention. At its end, the groups were crossed over, and all participating fellows received the refresher training program in the same academic year. We also hypothesized that our long-term CCUS educational effort would result in a notable learning transfer to clinical practice. We surveyed indications, types, and findings of CCUS examinations performed by fellows, along with the training program’s influence on patient care management.

## Methods

The study consisted of 1) a prospective randomized section to compare 2 training strategies (6-month ST vs IT) on improvement in CCUS proficiency and 2) a prospective section on CCUS examination surveys in the medical intensive care unit (MICU). It was conducted at the Mayo Clinic Hospital in Rochester, Minnesota, during the academic year July 2012 through June 2014. The Mayo Clinic Institutional Review Board approved the study (study ID 12–005,095).

### Comparison of 2 training strategies

Study participants were recruited among the entering critical care medicine fellows who underwent a multimedia CCUS workshop on 2 half-days in July 2012 or July 2013. This workshop was specifically designed for entering fellows as part of fellowship education. It consisted of didactic lectures and hands-on training. All 4 components of CCUS examination—cardiac, thoracic, abdominal, and vascular—were taught during the workshop***.***

All the participating fellows provided written informed consent to research enrollment. Then, the fellows took a baseline survey about their previous CCUS experience and a CCUS proficiency test within 2 weeks of the CCUS workshop. The proficiency test consisted of knowledge and skill portions. The knowledge portion measured participant knowledge on ultrasonography physics and image interpretation in 100 multiple-choice questions (maximum score, 100). Five questions were about machine knobology and ultrasonography physics; 10 questions were about vascular; 15 questions, thoracic; 55 questions, cardiac; and 15 questions, abdominal CCUS. The skill portion measured machine operation and image acquisition skills in simulated clinical scenarios (Additional file 1) with a maximum score of 100. This portion was video-recorded for subsequent scoring on 23 checklist items (Additional file 1) by 2 independent reviewers (R.S. and H.S.; scores by H.S. were used in the subsequent analysis). A total score of the CCUS proficiency test was calculated by combining the scores of knowledge and skill portions, with a maximum of 200 points.

Participants were first stratified into 2 groups on the basis of their total scores of the CCUS proficiency test, in high- (above the median) versus low-scoring groups. Subsequently, participants in each stratification group were randomly allocated into the ST or IT group.

The ST group received standardized CCUS education outlined in the Mayo Clinic critical care medicine fellowship education curriculum for 6 months—self-paced online learning modules, in-class lectures, and hands-on training at the bedside. The IT group received 8-h, additional training sessions during 1 of their 4-week MICU rotations. Several participants had 2 MICU rotations during the 6-month intervention. For these participants, 1 MICU rotation month was conveniently chosen as an IT month to avoid allocating too many learners in a particular month. Of the 8 h, the first hour was dedicated to performing CCUS examinations on a “standardized” patient, 6 h were spent at bedside examining MICU patients, and 1 h was spent for online image interpretation. The sessions were conducted hourly with prescheduled appointments, supervised by a staff intensivist or a certified sonographer according to their availability. The instructor to participant ratio was 1:1 in all training sessions.

At completion of the 6-month intervention, participants were asked to undergo the CCUS proficiency test. Contents were the same as the baseline CCUS proficiency test except for the order of the knowledge questions. After the 6 months, the group assignment was crossed over, and all learners initially in the ST group underwent additional 8-h training sessions as the IT group had done in the intervention period.

### CCUS examination survey

Study participants were recruited among the entering critical care medicine fellows who underwent a multimedia CCUS workshop on 2 half-days in July 2012 or July 2013. Study participants were asked to complete a survey each time they performed a CCUS examination in actual patient care in the MICU. The survey was intended to describe indications, types, and findings of CCUS examination performed by the participants, the influence of the examination on patient care management, and subjective confidence levels of the participants on image acquisition, interpretation, and overall management. The survey was collected from July 2012 through June 2014.

### Statistical analysis

In a prospective randomized study comparing 2 CCUS training strategies, no reasonable estimate of standard deviation (SD) of the CCUS proficiency test was available from the literature since the test was created specifically for this study. On the basis of previous similar CCUS education projects [18, 19], we anticipated that the SD would be approximately 6.5. For a clinically significant minimum improvement difference of 10 points between ST and IT groups, 8 participants in each group had 80% power to detect the difference in score improvements, with an α level of 0.05.

An association between the participant baseline ultrasonography experience and the group allocation was analyzed with Fisher exact test for each examination category. Quantitative parameters were expressed as median (interquartile range [IQR]), and Wilcoxon rank sum test was used to compare individual score improvements between the 2 groups. Improvement in test scores within each group was analyzed with Wilcoxon signed rank test. For scoring the skill portion of CCUS proficiency test, a Bland–Altman plot was used to analyze agreement between 2 independent reviewers (R.S. and H.S.). Only the participants able to complete both baseline and postintervention CCUS proficiency tests were included in the analysis.

In a prospective study on CCUS surveys, categorical variables such as examination type were presented with frequency and proportions. Binary variables, such as unexpected findings and change in management, were compared among types of CCUS examinations with χ^2^ test.

All statistical analyses were conducted with JMP Pro Version 10 software (SAS Institute Inc). *P* less than 0.05 (2-sided) was determined as statistically significant.

## Results

### Prospective randomized study comparing the 2 training strategies

A total of 32 fellows initially agreed to participate in the study. However, 11 fellows withdrew from the study because of scheduling conflicts or inability to complete the knowledge or skill portion, or both, of the CCUS proficiency test. Hence, 21 participants—10 in the ST group and 11 in the IT group—were included in the analysis. Table 1 summarizes the participants’ previous experience in point-of-care ultrasonographic examinations. Most fellows had 20 or fewer examinations in each category, and no statistically significant association was found between group allocation and prior ultrasonography experience (cardiac, *P* = 0.71; vascular, *P* = 0.67; thoracic, *P* = 0.42; and abdominal, *P* = 0.27).

**Table 1:** Participant Baseline Critical Care Ultrasonography Experience

Ultrasonographic Examination, No	Standard Training (*n* = 10)	Intensive Training (*n* = 11)	*P* Value
Cardiac			
0	4	5	.71
1–5	3	2	
6–10	1	2	
11–20	0	1	
21–50	2	0	
> 50	0	1	
Vascular			
0	5	5	.67
1–5	2	1	
6–10	2	2	
11–20	0	2	
21–50	1	0	
> 50	0	1	
Thoracic			
0	3	5	.42
1–5	3	4	
6–10	3	0	
11–20	1	2	
21–50	0	0	
> 50	0	0	
Abdominal			
0	3	5	.27
1–5	6	3	
6–10	0	0	
11–20	0	1	
21–50	1	0	
> 50	0	2	

**Fig. 1:**
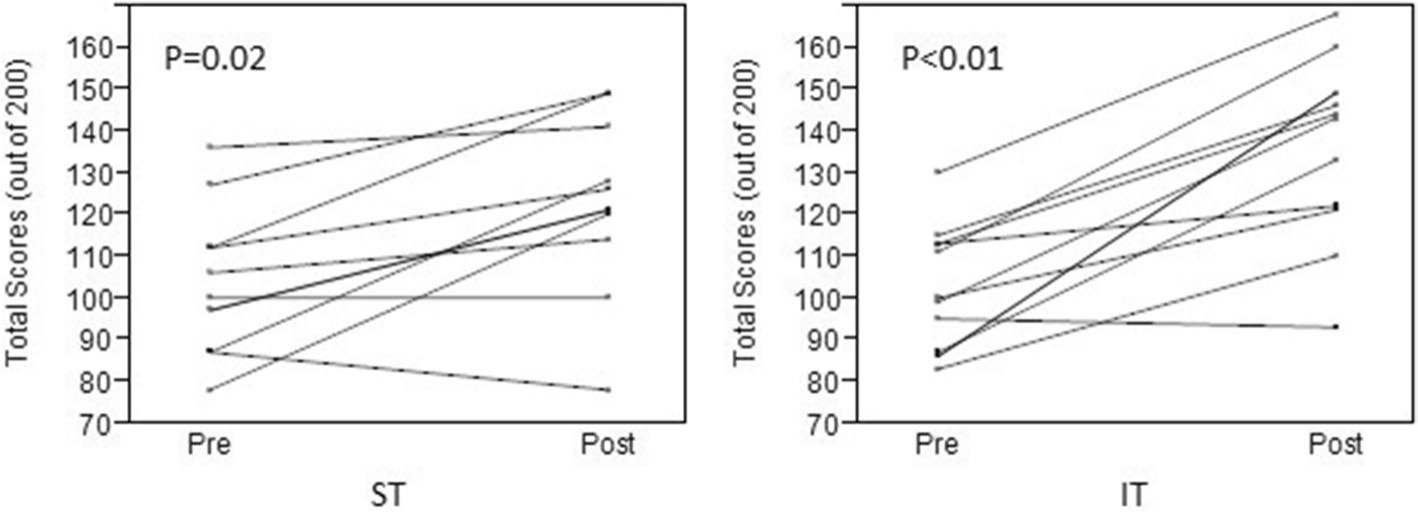
Individual Improvement in Critical Care Ultrasonographic Proficiency Scores of ST and IT Groups. IT indicates intensive training; pre, preintervention; post, postintervention; ST, standard training

**Fig. 2:**
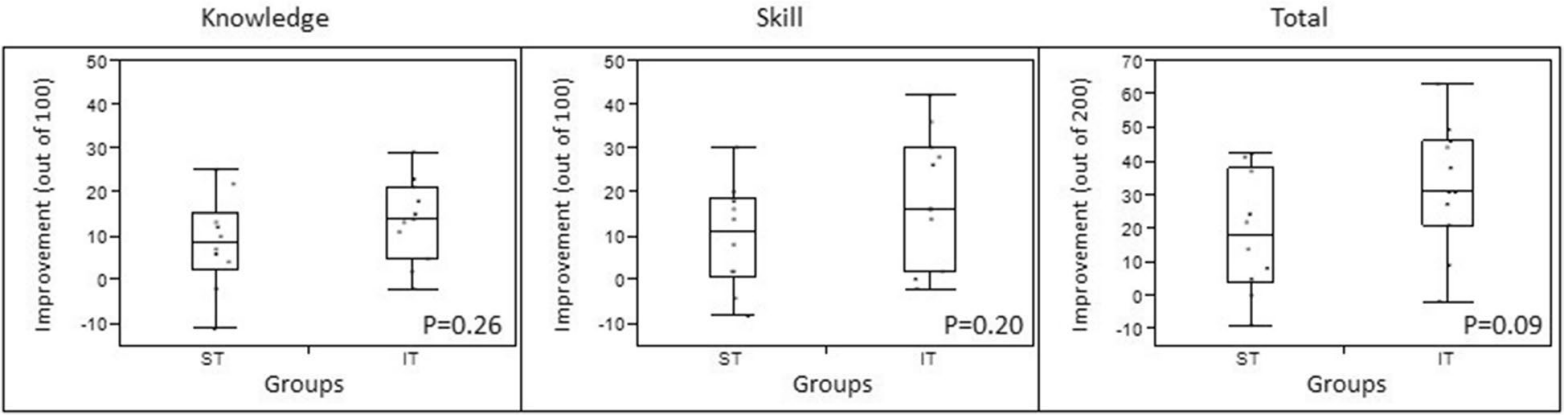
Improvement in Critical Care Ultrasound Proficiency Scores by Test Domain. Comparison was made between ST and IT training groups. IT indicates intensive training; ST, standard training

Table 2 shows test scores in the pre- and postintervention periods. No statistically significant difference was determined between the ST and IT groups in median total CCUS proficiency test scores in the preintervention period (*P* = 0.97). Figure 1 shows the individual improvement in total CCUS test scores at the end of the 6-month intervention. In this parameter as well, no statistically significant difference was observed between the 2 groups (Fig. 2): knowledge, median (IQR), 8.5 (2.5–15) versus 14 (5–21), *P* = 0.26; skill, 11 (0.5–19) versus 16 (2–30), *P* = 0.20; and total score, 18 (3.8–38) versus 31(21–46), *P* = 0.09.

**Table 2:** Critical Care Ultrasonography Proficiency Test Scores in Preintervention and Postintervention

	**Scores, Median (IQR)**		
**Period**^**a**^	**Standard Training (*n* = 10)**	**Intensive Training (*n* = 11)**	***P* Value**
Preintervention			
Knowledge	64 (57–71)	59 (51–69)	.40
Skill	39 (32–49)	44 (28–52)	.75
Total	103 (87–116)	100 (87–113)	.97
Postintervention			
Knowledge	72 (67–79)	80 (69–84)	.20
Skill	51 (43–63)	58 (52–70)	.11
Total	124 (111–143)	143 (121–149)	.22

*Abbreviation*: *IQR* Interquartile range

^a^ Maximum scores of knowledge period, 100; skill period, 100; and total, 200

**Fig. 3:**
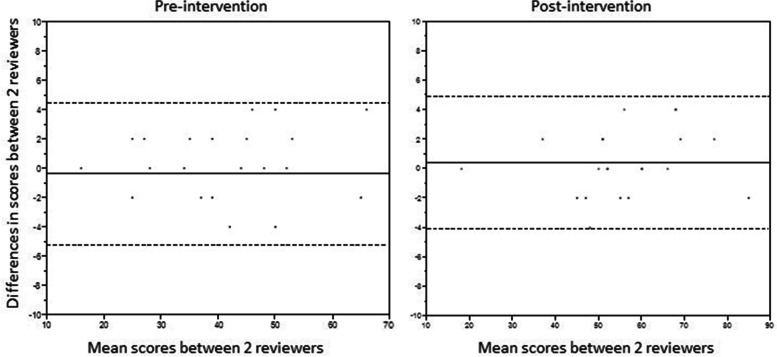
Bland–Altman Plots of Pre- and Postintervention Periods on Critical Care Ultrasonography Proficiency Test Scoring by 2 Independent Reviewers

Total CCUS test scores were significantly higher postintervention than preintervention in both groups (ST, 103 [87–116] vs 124 [111–143], *P* = 0.02; IT, 100 [87–113] vs 143 [121–149], *P* < 0.01). The Bland–Altman plot presented an acceptable level of agreement in scoring CCUS proficiency tests between the 2 reviewers (Fig. 3). Mean bias was − 0.38 with limits of agreement − 5.2 to 4.5 in the preintervention CCUS proficiency test; mean bias was 0.38 with limits of agreement − 4.1 to 4.9 in the postintervention CCUS proficiency test.

### Prospective study of CCUS examination survey

During the 2-year study, 38 fellows conducted 580 CCUS examinations, among which 400 examinations (69%) were properly documented in the survey. Among them, 226 examinations (57%) were performed by the 32 critical care medicine fellows who participated in the prospective randomized study comparing 2 training strategies. The common indications (including multiple indications per examination) were cardiovascular failure or shock (*n* = 272, 68%), respiratory failure or distress (*n* = 108, 27%), abdominal pain or discomfort (*n* = 30, 8%), and extremity abnormality (*n* = 27, 8%). Up to 73% of examinations (*n* = 292) were limited to 1 of these 4 common types of CCUS examination per patient, whereas the other examinations (*n* = 118, 30%) were combinations of those 4 types. Among single-type CCUS examinations, cardiac CCUS was most frequently conducted (*n* = 195, 67%), followed by thoracic (*n* = 50, 17%), abdominal (*n* = 33, 11%), and vascular CCUS (*n* = 14, 5%). Among combined CCUS examinations, the cardiac and thoracic combination was performed most commonly (34/118, 29%), followed by cardiac and abdominal (*n* = 27, 23%) and cardiac and vascular (*n* = 18, 15%).

Unexpected findings were seen in 120 examinations (30%)—most commonly seen in single cardiac exams (*n* = 52), followed by combined cardiac and thoracic (*n* = 13), single thoracic (*n* = 13), and combined cardiac and abdominal exams (*n* = 12). Patient care plan changed in 227 cases (57%) after CCUS examinations—95 (49%) among the single cardiac, 35 (70%) among single thoracic, and 21 (64%) among single abdominal. Median (IQR) confidence level on image acquisition was 8 (7–9) on a 10-point Likert scale. Median (IQR) confidence level on image interpretation was 8 (7–9). CCUS examination increased subjective confidence levels in patient care of 382 cases (96%).

## Discussion

Our study demonstrated that critical care medicine fellows have significantly improved CCUS proficiency in image acquisition and interpretation after a 6-month training intervention. This finding supports the effectiveness of the ultrasonography education program at our institution. However, no additional advantage was observed in IT group participants, who took an additional 8-h refresher training compared with the ST group. Many CCUS examinations were performed by fellows directly involved in the care of critically ill patients, which led to identification of unexpected findings, changes in patient management plans, and an increase in subjective confidence level in patient care.

This study represents the complexity of CCUS training, its optimal timing, and the contents of its reinforcement. Important for maximally effective training, a training program needs to be an integrated process rather than a discrete training opportunity [34–36]. A learning experience is maximized when the process is longitudinal, consisting of phases before, during, and after training [35]. We used a previously reported multimedia CCUS workshop as an initial introduction in the pretraining phase [18, 19]. This workshop was intended not only to enhance but also to standardize the learner’s baseline CCUS proficiency. The ST and IT programs were compared to pursue an optimal learning strategy during the training phase. Lastly, we conducted CCUS examination surveys to monitor learning transfer in the post-training phase.

The IT group took 8-h refresher sessions in addition to the CCUS education program outlined in our fellowship program. In general, a refresher training program is characterized by the type, frequency, and intensity of reinforcement [24]. Our refresher program was distinctive in that it was conducted in a month of MICU rotation, which occurred randomly during the study because of learners’ rotation schedules. Our study did not show a statistically significant difference in learner improvement between the ST and IT groups. However, it is difficult to conclude which components of reinforcement (type, frequency, and intensity) can be modified to further improve CCUS proficiency significantly beyond the level already attained through the fellowship education curriculum. Many academic societies have published training and practice guidelines on CCUS; those guidelines define CCUS education frameworks, such as the duration of the training and/or the number of proctored examinations to attain CCUS proficiency [7, 8, 13, 16, 37–40]. However, the methods to build a specific strategy to achieve those goals and incorporate it into the existing training curriculum are left to the discretion of each residency or fellowship training program. This is likely because every training program has a unique educational background with different teaching priorities. The literature remains scarce on the optimal components of CCUS education reinforcement, and the list of potential revisions is endless. Yet, we believe that the lessons learned from this study still remain valid and relevant to the current landscape of CCUS education, despite its rapidly evolving clinical practice over the last decade. This study revealed challenges in further enhancing a level of an existing education program and serves as a valuable resource to educators and learners who strive to improve CCUS education in a limited training period and finite resources.

Although we used a newly developed CCUS proficiency test as a surrogate to measure training effectiveness, the final goal of CCUS training is the use of CCUS examination in patient care. Through our study, we intended to observe learning transfer—to learn the extent of training that can be transferred to clinical work beyond 6 months of training strategy comparison. During 2 years of observation, 580 CCUS examinations (excluding central line insertion) were performed by critical care medicine fellows. CCUS resulted in a change of more than 50% in patient management plans, which is comparable to previous studies demonstrating 18% to 67% change in physicians’ management plans [41–43]. We do not have institutional data on the exact number of CCUS examinations before our study. However, given that most participants were novice learners of CCUS, this result suggests that our educational efforts successfully led to learning transfer.

Our study has several limitations. First, as many as 34% of fellows withdrew from the study, which may have caused a selection or attrition bias among participating fellows. This suggests that only the learners eager to improve their CCUS proficiency were able to complete the postintervention CCUS test or an additional refresher training if assigned in the IT group, or both the test and the training. This detail likely affected our study results, and the effect of our refresher program may have been underestimated since participants in the ST group may have been highly motivated at baseline. Second, participating fellows were not prevented from taking an ultrasonography-elective month or participating in an educational workshop outside our institution. Furthermore, our 8-h intervention could have impacted the quality and quantity of the other self-directed learning on CCUS after the study intervention. Unfortunately, we do not have data on the hours that each learner had spent on ultrasound education outside of the intervention, which may have affected our study results. Third, the timing of our refresher program differed among participants because it was conducted at the time of the learner’s MICU month. This likely affected learner scores of a CCUS proficiency test in the IT group. Fourth, the response rate of the CCUS survey was only 69%, and possibly learners completed the survey only when CCUS findings were critical or when patient care was changed, in which cases the clinical influence of CCUS may have been overestimated. Fifth, our CCUS proficiency test was designed specifically for this study and its content has not been validated. Various assessment modalities have been published over the last decade, and it is possible that the use of a different modality in assessing the intervention effectiveness might have produced a different interpretation of our study outcomes.

In conclusion, our study demonstrated that fellows in critical care medicine improved their CCUS proficiency significantly after a 6-month training program; however, the additional 8-h refresher training did not provide further benefit. Many CCUS examinations performed by fellows led to changes in patient care plans, a detail that suggests successful learning transfer in our educational program. The optimal type, frequency, or intensity of CCUS refresher training as part of continuous education is an important yet challenging theme to pursue for CCUS educators.

## Supplementary Information

**Additional file 1. **Skill Portion of the Critical Care Ultrasonography (CCUS) Proficiency Test.

## Data Availability

The datasets generated and/or analyzed during the current study are available from the corresponding author on reasonable request.

## Abbreviations

CCUS: Critical care ultrasonography
IT: Intensive training
IQR: Interquartile range
MICU: Medical intensive care unit
SD: Standard deviation
ST: Standard training
