# Supplementary material for: A prospective randomized study to compare standard versus intensive training strategies on long-term improvement in critical care ultrasonography proficiency
Source: BMC Med Educ. 2022 Oct 24;22:732. doi: 10.1186/s12909-022-03780-2 (PMC9594969; doi:10.1186/s12909-022-03780-2)
Supplement: Supplementary file 1 — Additional file 1. Skill Portion of the Critical Care Ultrasonography (CCUS) Proficiency Test. [file 12909_2022_3780_MOESM1_ESM.docx]

**Additional file 1.** Skill Portion of the Critical Care Ultrasonography (CCUS) Proficiency Test

**Scenarios**

Skill portion of the CCUS proficiency test consisted of 6 sections. An examinee was asked to complete the tasks on the written instruction sheets within a set timeframe. A “standardized” patient was a healthy male volunteer.  **Instructions:**

1. A target organ needs to be **in the** **center** of the screen with a correct orientation.
2. **Save** video images with appropriate depth and gain.
3. Video recording is set as a **6-second** **prospectively**. The machine will record a 6-second video clip **after** you press a button.
4. Examine the patient with **appropriate patient positioning**, except abdominal CCUS.
5. Do not switch off the machine until you are told to do so.

**Section 1: Starting the Examination**

You are going to **start a CCUS** examination.

You have **3 minutes** to complete your task.

1. Switch the machine on.
2. Manually enter or scan the patient’s clinic number to start the examination. (No need to enter the patient’s first and last name.)
3. When your task is completed, let the examiner know.

**Section 2: Vascular CCUS**

You are about to perform a **vascular CCUS** examination of a patient with a swollen right leg.

You have **4 minutes** to complete your task.

1. Visualize the **right common femoral vein and artery** (2 structures side by side). Make sure you have an appropriate depth and gain setting.
2. Save a video clip with a **compression maneuver**.
3. Perform a vascular CCUS examination to visualize the **right popliteal vein and artery**. Make sure you have an appropriate depth and gain setting.
4. Save a video clip with a **compression maneuver**.
5. When your task is completed, let the examiner know.

**Section 3: Abdominal CCUS**

You are about to perform an **abdominal CCUS** examination on a patient with hypotension, abdominal pain, and anuria. The patient is in supine position and **cannot move**.

You have **2 minutes** for probe switching and **4 minutes** to complete the following task.

1. Visualize the **abdominal aorta** and save a video clip in a **longitudinal view**.
2. Visualize the **left kidney** and save a video clip in a **longitudinal view**.
3. Visualize the **gallbladder** **body (fundus)** and save a video clip in a longitudinal view.
4. When your task is completed, let the examiner know.

**Section 4: Thoracic CCUS**

You are about to perform a **thoracic CCUS** examination on a patient with respiratory distress.

You have **3 minutes** to complete your task.

1. Perform a CCUS examination of the **right anterior chest** of the patient (**second or third intercostal space** in the midclavicular line) and save a video clip. Make sure you have an appropriate depth and gain setting.
2. Visualize the **entire** **right hemidiaphragm** and save a video clip. Make sure you have an appropriate depth and gain setting.
3. When your task is completed, let the examiner know.

**Section 5: Cardiac CCUS**

You are about to perform a **cardiac CCUS** examination on a hypotensive patient.

You have **2 minutes** for changing the examination mode and **6 minutes** to complete your task.

1. Obtain a **subcostal** (subxiphoid) **4 chamber view** and save a video clip.
2. Obtain a **subcostal** (subxiphoid) **inferior vena cava view** and save a video clip.
3. Obtain a **parasternal long-axis view** and save a video clip.
4. Obtain a **parasternal short-axis view** at the **papillary muscle level** and save a video clip.
5. Obtain a **parasternal short-axis view** at the **aortic valve level** and save a video clip.
6. Obtain an **apical 4-chamber view** (avoid foreshortening) and save a video clip.
7. When your task is completed, let the examiner know.

**Section 6: Ending the Examination**

You are going to **end the examination**.

You have **3 minutes** to complete your task.

1. Appropriately end the examination and switch off the machine.
2. Wipe ultrasound gel off of the patient.
3. Clean the machine and the probe.
4. When your task is completed, let the examiner know.

**Checklist**

The CCUS skill checklist included 23 items, with a maximum raw score of 50. The raw score was doubled to make the maximum score of 100, to match a score of knowledge portion (maximum score, 100). Knobology comprised 14% of a total score, while cardiac 38%, abdomen 18%, vascular 18%, and thoracic CCUS 12%. The entire skill test was videotaped, and the archived images were reviewed for subsequent scoring by 2 investigators (R.S. and H.S.). When a participant recorded multiple images for 1 task, the most preferable image was scored by reviewers.

For knobology and patient positioning, 1 point was given if a participant was able to complete the task. For organ visualization, no point was given for no images or unidentifiable structures, 1 point for an inadequate image of a target organ, which precludes from answering a clinical question, 2 points for a suboptimal image of a target structure, yet acceptable to answer a clinical question, and 3 points for an optimal image in every aspect, including depth and gain, and is good enough to answer a clinical question. A full 2 points were given for a complete compression maneuver; however, only 1 point was given for an inadequate compression.

| **Section** | **Clinical Questions** | **Checklist Items** | **Scores** |
| --- | --- | --- | --- |
| Knobology | None | 0.1 switch on the machine? | 1 |
|  |  | 0.2 start a new examination and enter clinic number? | 1 |
|  |  | 0.3 switch a transducer from vascular to abdomen? | 1 |
|  |  | 0.4 switch an examination mode from abdomen (thoracic examination) to cardiac? | 1 |
|  |  | 0.5 end the examination before switching of the machine? | 1 |
|  |  | 0.6 wipe ultrasound gel off from the standardized patient? | 1 |
|  |  | 0.7 clean the machine and transducers? | 1 |
| Thoracic | Pneumothorax or diaphragmatic dysfunction? | 1.1 visualize the anterior thorax?  1.2 visualize the right diaphragm? | 3  3 |
| Abdomen | Aortic aneurysm, hydronephrosis, or cholecystitis? | 2.1 visualize the aorta?  2.2 visualize the left kidney?  2.3 visualize the gallbladder? | 3  3  3 |
| Cardiac | Cardiomyopathy, valvulopathy, or abnormal central venous pressure? | 6.0 optimize patient position for the examination? | 1 |
|  |  | 6.1 obtain a subcostal 4-chamber view? | 3 |
|  |  | 6.2 obtain the inferior vena cava view? | 3 |
|  |  | 6.3 obtain the parasternal long axis view? | 3 |
|  |  | 6.4 obtain the parasternal short axis view at mid papillary level?  5.5 obtain the parasternal short axis view at aortic valve level?  6.6 obtain the apical 4-chamber view? | 3  3  3 |
| Vascular | Atherosclerosis or deep venous thrombosis? | 7.0 optimize patient position for the examination? | 1 |
|  |  | 7.1 visualize the right common femoral vein and artery? | 3 |
|  |  | 7.2 visualize the right popliteal vein and artery? | 3 |
|  |  | 7.3 preform compression maneuver? | 2 |
|  |  | Total | 50 |
